# The Voluntary Hospitals Commission on Hospital Management

**Published:** 1923-01

**Authors:** 


### THE VOLUNTARY HOSPITALS COMMISSION ON HOSPITAL MANAGEMENT.

THE Voluntary Hospitals Commission has received requests from several local hospital committees for some general suggestions as to the tests of efficiency in hospital management. In response to these requests, they have caused to be prepared a valuable pamphlet, "Notes on Hospital Management," from which we have made considerable extracts in the following paragraphs. The pamphlet itself may be obtained from the Stationery Office or the usual agents, at a cost of 3d. The Commission points out that it is impossible to lay down hard and fast rules applicable to all cases, since the term "hospital" covers a variety of institutions, ranging from the hospital with a medical school with 1,000 beds to the cottage hospital with 10 beds. The object has therefore been to summarise the principles which have been followed by the most experienced and successful hospital administrators; but it will be understood that there are few, if any, rules which may not require some modification in the light of local conditions. If any committee desires more detailed information on any specific point, the Commission will endeavour to procure it for them. Col. R. J. S. Simpson (late A.M.S.), C.B., C.M.G.; Mr. J. G. T. Buckle, Secretary, University College Hospital; Mr. E. W. Morris, C.B.E., House Governor, London Hospital; and Mr. R. Orde, House Governor, Royal Victoria Infirmary, Newcastle, co-operated in producing these notes.

It is pointed out that for the supervision of routine expenses, often individually small, but amounting in the year to something considerable, it is well for the committee to include a member with knowledge of the details of catering and management, analogous to that of a managing director of a company. In any case in a large hospital one of the staff should possess some special qualifications of this kind. The committee should see that supervision of all details of expenditure is carried out by this officer.

Every hospit al must have access to some laboratory or X-ray clinic. Whether or not it has a laboratory of its own and an X-ray outfit depends on its number and class of patients and on the other facilities available. Where different hospitals are conveniently situated in relation to one another, the work and expense may be distributed between them. The smaller hospitals would be well advised to defer adopting new and costly equipment until its value has been established. Every effort should be made to secure the co-operation of the medical staff in the control of expenditure, and the risk of friction between them and the committee will be largely overcome if they are represented on the house and finance committee and other sub-committees and so brought into direct touch with revenue and expenditure.

A system of rationing departments is valuable, if only as a moral factor, in keeping down expenditure. Every hospital should prepare an annual budget, definite provision being made for all the services of the ensuing year; if the cost of any service or of any department exceeds the allocated sum, the cause should be investigated.

Cost per occupied bed is usually taken as the criterion in comparing hospitals with each other, and if they are properly classified according to size and class of work, with due regard to situation, it is not impossible to calculate what they should cost undef economical and efficient management. A system or uniform accounts suitable for each class of hospital, such as the Commission hope to produce shortly, should prove valuable.

Stress should be laid on the necessity of measuring the expenditure month by month of all articles consumed (food, drugs, dressings, coal and gas, &c.) in terms of the number of patients and of resident staff. Experience has proved the benefit of exhibiting to the members of the staff, both medical and nursing, comparative statements, showing each month the wards or other units in order of increasing expenditure.

In all hospitals someone must be responsible for the supervision of the expenditure in all departments. In the larger ones there is the superintendent or the secretary. Only in very small hospitals should the matron be the responsible authority or administrator. In medium or small hospitals the difficulty is greatest, but all expenditure should be controlled by a specially qualified administrator who should be (except in very small institutions) distinct from the technical and scientific staff.

### METHODS OF CONTROL.

King Edward's Hospital Fund enumerates the following heads as items of expenditure in their accounts:—

A. MAINTENANCE:
  1. Provisions.
  2. Surgery and Dispensary.
  3. Domestic.
  4. Establishment.
  5. Salaries, Wages, &c.
  6. Miscellaneous.

B. ADMINISTRATION:
  1. Management.
  2. Finance.

C. RENT, RATES AND TAXES.

D. EXTRAORDINARY EXPENDITURE.

In 1920 in the London area, the first three sections (Provisions, Surgery and Dispensary, and Domestic) accounted for 67 per cent. of the total cost per occupied bed in eleven large general hospitals with medical schools; for 72 per cent. in eight without medical schools; and for 71 per cent. in thirteen smaller general hospitals.

### PROVISIONS.

These include supplies for (*a*) patients; (*b*) staff. In both cases the diet must be adequate, without waste. The following scales, A. and B., have been found adequate, and, properly used, they allow sufficient variety. Scale A. was designed for bed cases ; Scale B. for convalescent cases, or for patients who required feeding up. Scale B. should also be sufficient for the female staff. It is important to note that these scales are per 100 patients, and are not intended to give by division a diet for a single patient. They automatically allow for the difference in appetite between individuals. They do not include any margin for avoidable waste, and their satisfactory use demands skilled and intelligent management and adequate cooking. They are intended merely as a check on the total consumption, and include articles which would not form part of the normal dietary and ought only to be given on special instruction from the medical officer concerned.

### Scales of Quantities Sufficient to Feed 100 Patients for One Day.

| | Scale A. | Scale B. |
|---|---|---|
| Meat | 31¼ lb. | 31¼ lb. |
| Fish | 18¾ lb. | 18¾ lb. |
| Bacon | 12½ lb. | 12½ lb. |
| Bread and Flour | 69 lb. | 75 lb. |
| Sugar | 9 lb. | 9½ lb. |
| Edible Fat (Butter, Margarine, Dripping) | 6½ lb. | 6½ lb. |
| Potatoes | 50 lb. | 70 lb. |
| Fresh Vegetables | 25 lb. | 35 lb. |
| Cocoa | 1 lb. | 1 lb. |
| Milk | 80 pts. | 130 pts. |
| Syrup and Jam | 5 lb. | 5 lb. |
| Cereals | 7 lb. | 20 lb. |
| Eggs | 40 | 50 |
| Tea and Coffee | 1½ lb. | 2 lb. |
| Cheese | 3 lb. | 3 lb. |
| Approximate calorie value per patient | 2,700 | 3,250 |

The following dietary scales, which are in use in a London hospital of medium size with a medical school, are appended for purposes of comparison :—

| *Ordinary Diet.* | *Spoon Diet.* | *Strict Spoon Diet.* |
|---|---|---|
| 10 oz. Bread. | 10 oz. Bread. | 3 pts. Milk. |
| 5 oz. Potatoes. | 2 pts. Milk. | 1 pt. Beef Tea. |
| 5 oz. Green Vegetables. | 1 pt. Beef Tea. | |
| *4 oz. Meat (Males). | 2 oz. Butter. | |
| *3 oz. Meat (Females). | Custard Pudding. | |
| *or* | | |
| 8 oz. Fish | | |
| ½ pt. Beef Tea. | | |
| Milk Pudding. | | |
| (Suet pudding and treacle one day per week.) | | |
| 1 pt. Milk. | | |
| 1 oz. Butter. | | |

Menu of Meat :

*Monday* and *Saturday,* Roast Mutton ; *Sunday, Tuesday* and *Thursday,* Roast Beef ; *Wednesday,* Boiled Mutton ; *Friday* Fish.

Extras ordered by a member of the medical staff for a limited period only :—

| | |
|---|---|
| Wines, Spirits, Malt Liquors. | Eggs |
| Cream. | Stewed Fruits. |
| Meat Juice. | Lemons. |
| Bacon. | Jellies. |
| Chickens. | Rusks. |
| Rabbits. | Diabetic Bread, &c., &c. |

* Cooked meat.

In the event of any district desiring to standardise its dietary, a comparison of the actual diets in use with the above table may prove of value.

### Surgery and Dispensary.

*Drugs.*—Stock mixtures should be used as far as possible ; they save time, and reduce the stock of drugs to be maintained. Formulæ may often be revised with advantage. Many were written a good many years ago ; advances in pharmacology and improvements in trade preparations have now made it possible to obtain an equivalent combination at less than the cost of the original. A trained pharmacologist (a medical man) should be consulted on this point, and might also be asked to draw up a list of expensive drugs which should not be used except by special order.

*Dressings and Bandages.*—Ward account books should be kept showing quantities used. Much avoidable expenditure could be eliminated if attention were called to this waste. Publication of a list of wards in order of least expenditure is beneficial. Cost and saving should be shown. Similar lists should be prepared for operating theatres and medical wards.

### Domestic.

*Breakages* always form an important item, and the great expenditure incurred by breakage is often not sufficiently impressed on the staff. A record of breakages should be kept, and a comparative statement, unit by unit, issued.

*Washing.*—Whether the hospital should have a laundry or send its washing out must be decided individually, but the amount of washing dealt with is important. In 11 London hospitals in 1916 the average number of pieces per week per patient was 21. In the same hospital in 1917 it was 12—an economy of 43 per cent., amounting to a saving of £21,000 per annum, taking the cost of washing at 1d. per article. In seven London hospitals the number of pieces per bed washed per week varied between 20 and 65—with an average of 37 per bed per week.

*Water.*—Consumption is unavoidably large, especially where there is much corridor space. But if it is checked by frequent readings of the meter, and waste prevented by supervision of all taps and pipes, and by limitation of the amount used in baths (each bath should have a line showing the upper level of water to be used), some reduction will usually be possible.

*Fuel and Lighting.*—Waste is frequently due to the use of sources and apparatus of low efficiency, or to faulty employment of a relatively efficient apparatus. The multiplication of sources increases waste. If heat for warming, cooking and the supply of hot water for domestic use, including washing, can be drawn from one source, waste is diminished. A system of heating through radiators by low pressure steam, produced by a single system of boilers, cooking (*i.e.*, boiling and the like) in steam-jacketed vessels, and the heating of water by the waste gases, tends to make waste as small as possible. Incandescent gas lighting appears usually to be cheaper than electricity. But the enormous advantage of electricity outweighs its greater cost. In any case, in a modern hospital, it is almost essential to have current for other purposes than lighting. Power and lighting current must be distinguished and measured and recorded separately. The secret of control of consumption is the multiplication of meters, their periodical reading (once a day is not too often), and comparison and publication of records not less than once a month. Staff quarters, kitchen, stores, and wards (at least each block of wards) should have their own meters.

The following figures, which represent the consumption of one of the London hospitals with medical school, may be useful for purposes of comparison :—

| | |
|---|---|
| Coal .. .. | 7-8 tons per bed per year. |
| Gas .. .. | 18,000-20,000 cubic feet per bed per year. |
| Electric light .. | 80-100 units per bed per year. |

SALARIES, WAGES, &C.

The proportion of the total cost per bed under this head was 32 per cent. in the large general hospitals with medical schools, 27 per cent. in those without medical schools, and 26 per cent. in the smaller general hospitals (not including salaries of officials or pensions.

SOME GENERAL CONCLUSIONS.

Comparatively small and relatively inexpensive alterations may make the working of a department much easier, and so economise time and labour, and therefore money. This applies particularly to the kitchen department (for example, in the substitution of gas cookers for coal ranges, the arrangements for reception and storage of articles of food) and to the issue of meals. The collection and disposal of soiled linen and the distribution of clean are laborious operations which can be made easier by adequate conveniences. If the principle of the economy of time and labour be borne in mind, much may be done by re-organisation, with or without minor structural alterations.

The system of control by record and periodical comparison of expenditure is useless unless some authority, outside the spending departments, takes an active and intelligent interest in these records. Their preparation is useless expense unless they are scrutinised and action taken on the results shown.

